# StethAid: A Digital Auscultation Platform for Pediatrics

**DOI:** 10.3390/s23125750

**Published:** 2023-06-20

**Authors:** Youness Arjoune, Trong N. Nguyen, Tyler Salvador, Anha Telluri, Jonathan C. Schroeder, Robert L. Geggel, Joseph W. May, Dinesh K. Pillai, Stephen J. Teach, Shilpa J. Patel, Robin W. Doroshow, Raj Shekhar

**Affiliations:** 1 Sheikh Zayed Institute for Pediatric Surgical Innovation, Children’s National Hospital, Washington, DC 20010, USA; 2 AusculTech Dx, 2601 University Blvd West #301, Silver Spring, MD 20902, USA; 3 School of Medicine and Health Sciences, George Washington University, Washington, DC 20052, USA; 4 Division of Pulmonary and Sleep Medicine, Children’s National Hospital, Washington, DC 20010, USA; 5 Department of Cardiology, Boston Children’s Hospital, Boston, MA 02115, USA; 6 Department of Pediatrics, Walter Reed National Military Medical Center, Bethesda, MD 20814, USA; 7 Department of Pediatrics, Children’s National Hospital, Washington, DC 20010, USA; 8 Division of Emergency Medicine, Children’s National Hospital, Washington, DC 20010, USA; 9 Department of Cardiology, Children’s National Hospital, Washington, DC 20010, USA

**Keywords:** digital stethoscope, pediatric stethoscope, artificial intelligence-assisted auscultation, Still’s murmur detection, wheeze detection, deep learning

## Abstract

(1) Background: Mastery of auscultation can be challenging for many healthcare providers. Artificial intelligence (AI)-powered digital support is emerging as an aid to assist with the interpretation of auscultated sounds. A few AI-augmented digital stethoscopes exist but none are dedicated to pediatrics. Our goal was to develop a digital auscultation platform for pediatric medicine. (2) Methods: We developed StethAid—a digital platform for artificial intelligence-assisted auscultation and telehealth in pediatrics—that consists of a wireless digital stethoscope, mobile applications, customized patient-provider portals, and deep learning algorithms. To validate the StethAid platform, we characterized our stethoscope and used the platform in two clinical applications: (1) Still’s murmur identification and (2) wheeze detection. The platform has been deployed in four children’s medical centers to build the first and largest pediatric cardiopulmonary datasets, to our knowledge. We have trained and tested deep-learning models using these datasets. (3) Results: The frequency response of the StethAid stethoscope was comparable to those of the commercially available Eko Core, Thinklabs One, and Littman 3200 stethoscopes. The labels provided by our expert physician offline were in concordance with the labels of providers at the bedside using their acoustic stethoscopes for 79.3% of lungs cases and 98.3% of heart cases. Our deep learning algorithms achieved high sensitivity and specificity for both Still’s murmur identification (sensitivity of 91.9% and specificity of 92.6%) and wheeze detection (sensitivity of 83.7% and specificity of 84.4%). (4) Conclusions: Our team has created a technically and clinically validated pediatric digital AI-enabled auscultation platform. Use of our platform could improve efficacy and efficiency of clinical care for pediatric patients, reduce parental anxiety, and result in cost savings.

## 1. Introduction

The traditional acoustic stethoscope, invented by Laennec in 1816 [1], remains a widely used screening tool among healthcare professionals [2,3]. Over the past 200 years, it has undergone many refinements such as the introduction of a tunable diaphragm and double-lumen tubing design. However, the low intensity of the conducted sound makes auscultation difficult for many of its users. Acoustic engineers have improved the sound quality and amplification of the acoustic stethoscope, but with limited success [4]. Physicians often perform auscultation in noisy environments, and acoustic stethoscopes do not feature active noise cancellation. Acoustic stethoscopes also produce an irregular frequency response, distorting or suppressing the high-frequency components of some cardiopulmonary conditions [5]. As a result, some cardiopulmonary conditions may go undetected by primary care providers (PCPs) [6].

Digital stethoscopes were developed as a superior alternative because they could deliver sound amplification and a more consistent frequency response while also providing active noise cancellation. They can further function as a useful tool for teaching auscultation because of the ease of making recordings and their high quality. Some small heart and lung sound libraries have been created using digital stethoscopes to teach students the art of auscultation [7]. Nevertheless, mastering this skill can be challenging and often requires extensive training. In a systematic review of published papers on auscultation between 1967 and 2021, Herefoss et al. reported that the sensitivity and specificity of auscultation varied greatly among physicians [8]. For example, for valvular heart disease, the sensitivity of auscultation ranged from 30% to 100%, whereas the specificity ranged from 28% to 100% [8]. Having high sensitivity and specificity among all physicians is indeed highly desired.

To tackle this challenge, researchers are examining the use of artificial intelligence (AI) to help clinicians interpret auscultated sounds. However, a paucity of datasets in pediatric cardiology and pulmonology represents a significant limiting factor [9]. To date, the clinical adoption of AI-assisted auscultation is still lacking, and the use of digital stethoscopes and AI remains in its early stages of development. Nevertheless, researchers anticipate a transformation in the use of auscultation, all this while preserving conventional and simple workflows, with greater levels of consistency and accuracy.

**Figure 1:**
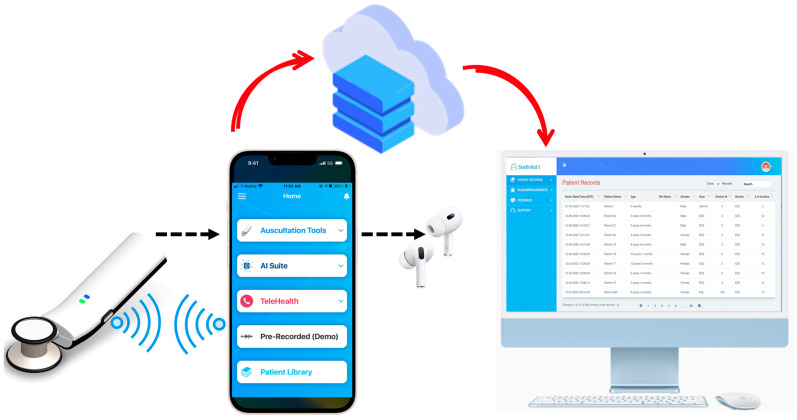
StethAid platform.

To the best of our knowledge, there is currently no AI-based digital auscultation platform that is specifically tailored for pediatrics. We present here StethAid, a platform that consists of a digital stethoscope, a mobile application, custom cloud storage, and web-based portals and dashboards (see Figure 1). A custom digital stethoscope connects to the custom mobile app via Bluetooth technology. The stethoscope features both active noise cancellation and digital filtering and supports both wired (USB-C) and wireless charging. The mobile app features streaming and recording of heart and lung sounds, thus allowing the building of patient sound libraries. Cardiologists and pulmonologists on our team access recordings offline using the web portals to review the quality of recordings and label sounds with clinical diagnoses.

The StethAid platform features three pediatric applications. The first application, StillsAI, is a deep learning-based decision support system intended for PCPs to identify the innocent Still’s murmur in children [10,11]. The Still’s murmur is the most common innocent murmur of childhood, and innocent heart murmurs are over 50 times more prevalent than pathological murmurs [12]. In the United States, an estimated 400,000 children per year are referred to pediatric cardiologists for the evaluation of a heart murmur. The Still’s murmur is the most common finding by pediatric cardiologists in this referred group. StillsAI would provide a PCP with real-time decision support, potentially reducing a large proportion of ultimately unnecessary referrals to the cardiologist for evaluation of a heart murmur [12].

The second use case of StethAid is for applying data-efficient deep learning models for automated wheeze detection in children to empower parents to assess the severity of their child’s asthma at home. Asthma is the most common chronic pediatric disease in the United States [13], affecting approximately 6 million, or 1 in 12, children. Early symptoms of asthma exacerbation are often nonspecific and are not recognized by parents until the child shows severe symptoms requiring emergent care. A caregiver’s task of assessing asthma severity is inherently difficult because it is subjective, often overlapping with common cold symptoms, and there are few objective tools for it. A key requirement for developing a mobile application for objective home management of acute asthma is automated detection of wheeze.

Our third application is telehealth. Providers are increasingly utilizing telehealth in daily clinical practice. Prior to 2020, telehealth services were growing, but the coronavirus disease 2019 (COVID-19) pandemic accelerated their adoption and continues to be widely employed [14]. For providers seeing recurring patients with chronic respiratory diseases, such as asthma, remote auscultation of the lungs can aid in distress situations in which the disease is exacerbated. The long-term care of these patients is shifting to telehealth visits because of their ease and flexibility; however, remote auscultation is still lacking because of technical limitations. The telehealth feature of StethAid is designed to meet this need of pediatric medicine. 

The rest of this paper is structured as follows: Section 2 describes a review of existing digital stethoscopes. Section 3 presents the material and methodology. Section 4 presents the frequency response of StethAid stethoscope and two emerging AI-based use cases of our digital auscultation platform. Section 5 and Section 6 summarize and conclude the paper.

## 2. Related Work

Several digital stethoscopes exist [4,15], though only a few could provide an AI-assisted interpretation of the digital recordings to aid physicians in making a diagnosis. Furthermore, these platforms generally focus on adult auscultation. In this section, we review common digital stethoscopes.

### 2.1. 3M Littmann 3200

3M Littmann 3200 (3M Company, St Paul, MN, USA) is a popular electronic stethoscope. It uses a piezoelectric sensor. It is equipped with a tunable diaphragm, stem, chestpiece, tubing, aerospace aluminum alloy eartube, headset, and snap-tight eartips [16]. Littmann 3200 provides primary ambient noise reduction and frictional noise dampening using dual-lumen tubing where the two sound paths are contained inside an outer tube to eliminate the rubbing noise. It features three auscultation modes: bell, diaphragm, and extended. The device supports 12 on-device recording tracks. The recordings can be imported to the companion StethAssist software. The sampling rate is 4 kHz [17]. The device’s sound level can be acoustically amplified. Littmann 3200 is powered using a AA alkaline battery that generally provides 50–60 h of continuous use, depending on the frequency of use. Littmann 3200 does not provide any software to assist in the identification of heart murmurs or specific lung sounds and can be used only as an electronic stethoscope. Littmann 3200 could be used to collect heart and lung sounds, but it is less practical for building large databases because of the limited number of recording tracks. 

### 2.2. Stethee Pro

Stethee Pro (M3dicine, Queensland, Australia) is an AI-powered wireless stethoscope designed for detecting cardiac and respiratory events [18]. The device includes five auscultation modes—bell, diaphragm, heart, mid-range, and extended—and can be used to auscultate body sounds such as those of the heart, lungs, and limbs. The reported frequency range is 20–20,000 Hz, but the specific frequency bands of the individual modes are not available. It can amplify sound up to 96 levels. The aerospace-grade aluminum alloy housing helps cancel out unwanted noise, while the rechargeable lithium-ion battery ensures mobility. With the Stethee Pro iOS app and Stethee Pro Android app, users can measure the durations of systole and diastole, heart rate, and respiratory rate. Additionally, the device can be used with either wired or wireless headphones. Stethee Pro also offers a central web application for data access and management. The device measures 1.6 inches × 2.4 inches (40 mm × 60 mm) and weighs 6 ounces (170 g).

### 2.3. Feelix/Feelix Pro

Feelix and FeelixPro (Sonavi Labs, Baltimore, MD, USA) are digital stethoscopes that serve as medical instruments to stream, amplify, and record sounds from the heart, lungs, abdomen, neck, limbs, arteries, veins, and other internal organs with selective frequency ranges [19]. The Feelix stethoscope is built using a microphone array to perform adaptive noise cancellation and does not include any rubber tubing. With the ability to store up to 50 recordings of 10 s each, the Feelix stethoscope features on-device recording and allows for 25 levels of sound amplification. Powered by a lithium-ion battery, the Feelix stethoscope supports both wired and wireless charging, providing approximately 3 h of continuous use on a single charge, according to the manufacturer. Sounds can be heard in real time using a Bluetooth-enabled or wired headset. The FeelixPro stethoscope gives users access to web portals and pairs with two iOS mobile apps, Feelix-Patient and Feelix-Provider, via Bluetooth. These apps enable the calculation of heart rate and respiratory rate and support data analytics to detect wheeze.

### 2.4. Eko Core

Eko Core (Eko Health, Oakland, CA, USA) is a hybrid stethoscope that users can toggle between analog (acoustic) and digital [20]. Eko Core stethoscopes have a similar construction to the Littmann 3200. Eko Core has three operation modes: cardiac, pulmonary, and wideband. The stethoscope does not support on-device recording. Eko Core can be linked with Eko mobile application for recording and saving the body sounds to the cloud. The sampling rate of the recorded audio files is 4 kHz. It supports wireless listening. Eko Core comes with a lithium-ion battery that needs about 2.5 h to be fully charged and provides about 8 h of continuous use. In a typical clinical setting, this represents about two weeks of use between charges. Eko Core provides up to 40 levels of sound amplification and supports AI for heart murmur detection with Eko analysis software.

### 2.5. Thinklabs One

The Thinklabs One digital stethoscope (Thinklabs Medical LLC, Centennial, CO, USA) has a different construction compared with Littmann 3200 and Eko Core and is similar to Feelix and Stethee Pro because of its tubing-free design. The Thinklabs stethoscope uses a capacitive transducer [21]. This stethoscope is made with chrome-plated aluminum for primary ambient noise reduction. It supports three major modes—bell: Filter 1 (30–500 Hz) and Filter 2 (60–500 Hz), diaphragm: Filter 3 (80–500 Hz) and Filter 4 (100–1000 Hz), and extended: Filter 5 (20–2000 Hz). Thinklabs One has more filtering options compared with Littmann 3200 and Eko Core as it offers two variations on the bell and diaphragm modes. It supports up to 100-fold sound amplification, according to the device specifications. Thinklabs One is interfaced with its companion ThinkLink software. Thinklabs One includes a rechargeable energy battery source and requires charging one to two times a week, supporting 100–125 patients per charge. Thinklabs One provides access to the eMurmur (CSD Labs, Graz, Austria) software for murmur diagnosis.

### 2.6. ViScope

The ViScope digital stethoscope (HD Medical, Sunnyvale, CA, USA) provides “Dynamic Auscultation” via an integrated phonocardiogram visual on an LCD screen [22]. The rest of ViScope’s construction is like that of an acoustic stethoscope, such as tubing, earpieces, and headset. ViScope also supports three modes: bell (20–300 Hz), diaphragm (40–500 Hz), and wide/extended range (40–800 Hz). ViScope features ambient noise-reduction technology. ViScope provides storage for up to four 10 s patient waveforms for documentation. It interfaces with a PC through the ViScope MD software, which allows for capturing, documenting, and archiving patient records. It has a tunable filter and supports an external amplifier. ViScope amplifies sounds up to 30 times louder with real-time digital filtering and adjustable volume control. The ViScope stethoscope is powered using a lithium-ion rechargeable battery that provides acceptable life between charges. ViScope MD has an embedded algorithm that enables screening for heart anomalies and murmurs. Additionally, ViScope MD supports an external amplifier for observation and discussion such as in teaching or consulting situations.

## 3. Material and Methods

This section describes the StethAid platform. First, we describe the design and development of the StethAid digital stethoscope. We describe the block diagram, the hardware usability, wireless connectivity, sound transduction, power feature, 3D printing and assembly, active noise cancellation, mobile applications, and web portals.

### 3.1. Block Diagram

**Figure 2:**
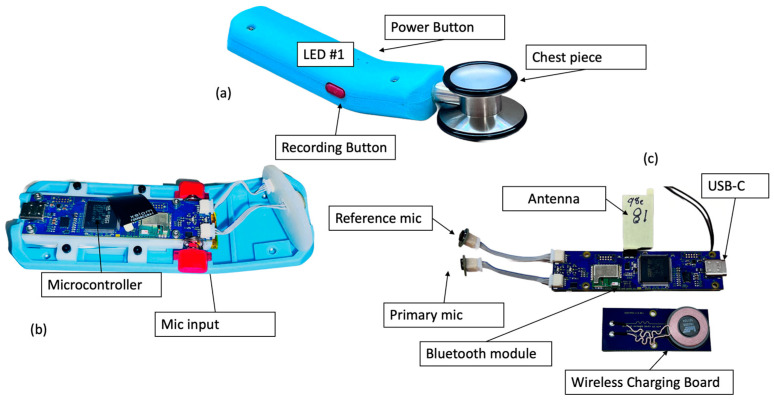
StethAid stethoscope: (**a**) assembled unit; (**b**) mic inputs and microcontroller; and (**c**) main and charging boards.

The block diagram of the StethAid digital stethoscope (Figure 2) consists of two MEMS microphones connected to the main printed circuit board (PCB). The block diagram includes light-emitting diodes (LEDs) and buttons, a Bluetooth module, a battery, and an accelerometer. The digital stethoscope supports wireless connectivity and wireless charging. The battery is a lithium-ion polymer battery. The firmware runs on a microcontroller.

### 3.2. Hardware Usability

**Figure 3:**
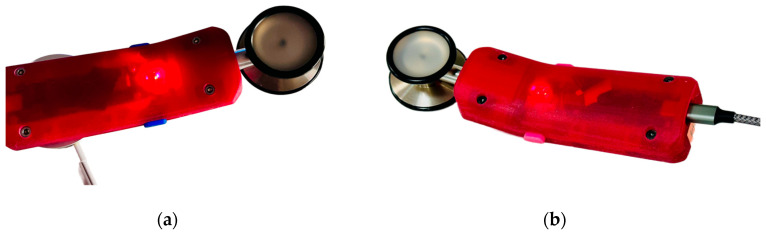
Charging of an assembled StethAid device: (**a**) wireless charging and (**b**) wired charging.

Through extensive user testing with clinicians, we found a user-friendly design for the device enclosure that is comfortable in the users’ right or left hand and suits various hand sizes (Figure 3). Iterating through clinical feedback, we have further refined the StethAid hardware for easy clinical usability by modifying power management states, adjusting the tactile feel of the 2 buttons, and modifying the LED indicators. The 2 LEDs on the device provide information regarding the current system state (ON/OFF, battery status, Bluetooth connectivity, and recording status) and mirror the primary functions that occur in the mobile app. We have put two physical buttons on the device to facilitate power and recording functionalities. These buttons can be mapped to multiple functions within the mobile application, but, through user testing, we found the recording and power functionalities to be the most useful. The recording button is tied to the recording functionality in the mobile application—this allows the user to easily record by pressing the button without having to touch the mobile device. 

### 3.3. Wireless Communication

The StethAid stethoscope utilizes a custom app to transfer the auscultated sound via Bluetooth to a mobile phone. The app can only be used if a registered digital stethoscope is paired. When a StethAid stethoscope is ready to be paired, LED#1 flashes blue and, when paired, it becomes solid blue. To pair the device, the user clicks on “Connect to Device” and picks the Hardware ID of the StethAid stethoscope to which the app is going to be connected. Once paired, the StethAid stethoscope wirelessly streams auscultated sounds, and a wireless or wired headphone set can be used to listen to them. Wireless listening makes using the StethAid stethoscope more convenient with better mobility.

### 3.4. Sound Transduction Pathway

The StethAid stethoscope uses a traditional stethoscope chestpiece combined with custom digital electronics and enclosure. StethAid uses two microphones that sample sound in the pulse density modulation (PDM) format at 1 MHz, and the sound signal is then converted into a pulse code modulation (PCM) format sampled at 8000 Hz. Heart and lung sounds range primarily between 20 and 2000 Hz; thus, a sampling rate of 8000 Hz is well in the range of adequate frequency for the present cardiac and pulmonary applications. The housing of the microphones has been optimized for friction noise reduction.

### 3.5. Power Features

The StethAid stethoscope features a rechargeable battery that lasts up to 5 days under normal use, defined as 30 min per day, or three patient visits averaging 10 min each. The device can be charged through either a USB-C cable or Qi induction wireless charging, as shown in Figure 3. The Qi coil is shown in Figure 2c. Qi wireless charging allows charging of the device by simply placing it on a charging station. The device charges to full capacity using Qi charging in approximately 2 h. When the device is charging, the LED#2 flashes an orange color and when it is fully charged it turns solid orange. At any moment, the level of the battery is shown on the setting menu of the StethAid apps. To conserve power, the device powers off automatically if it is not connected to a mobile phone or is idle for 5 min. When the StethAid unit is powered off, it can be turned on by pressing the power ON button or by picking it up and shaking it (the shake-to-wake feature). We have found power performance to be a critical feature for enhancing clinical usability of our device and have optimized it through clinical feedback. 

### 3.6. Three-Dimentional Printing 

**Figure 4:**
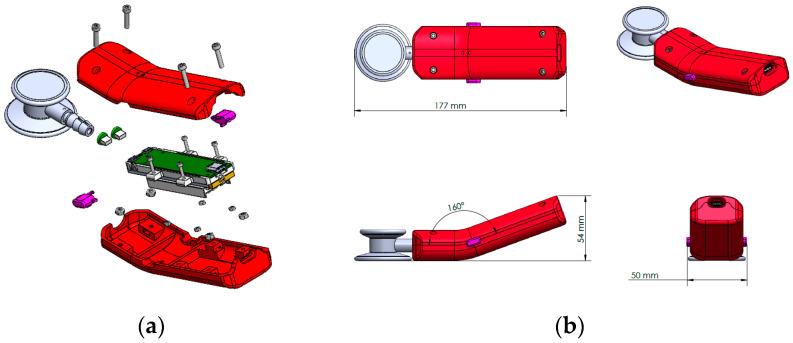
StethAid device (**a**) exploded view; (**b**) StethAid length, height, and width [23].

The stethoscope parts were designed in SolidWorks and optimized for 3D printing on the Stratasys J5 MediJet 3D printer [23]. The J5 supports multi-material and multi-color printing, with water-soluble support. These capabilities include transparent materials and flexible materials ranging from shore 50A to rigid. For completely rigid materials, the dimensional accuracy of the printer is ±0.15% of the part length. The build tray has a printing area of 1174 cm^2^ and maximum part size of up to 140 mm × 200 mm × 190 mm (5.51 inches × 7.87 inches × 7.48 inches). The J5 MediJet’s large tray accommodates multiple StethAid models in a single print, and the water-soluble support minimizes post processing labor. The StethAid exploded view is shown in Figure 4a. The maximum dimensions of the StethAid stethoscope are 177 mm (length), 48 mm (width), and 54 mm (height) (see Figure 4b). The StethAid stethoscope weighs 207.33 g.

For assembly simplicity and durability, pockets are 3D printed for the location of standard nuts. Utilizing the printer’s ability to print multiple materials, clear slots are printed directly into the outer case to focus the PCB’s LED indicators. For improved haptics of pressing the PCB’s on-board buttons, conical compression springs are press fit into printed holes. The printing time of a single StethAid unit is about 6 h. Utilizing the entire build tray, three units can be printed in about 14 h. 

### 3.7. StethAid Active Noise Cancellation

StethAid performs active noise cancellation (ANC) using two microphone and frequency-domain adaptive filtering in the firmware. The algorithm cancels unwanted noise from the heart or lung sounds. This feature can be used when the StethAid users are performing auscultation in noisy environments where the sirens and people conversing can contaminate the desired signal, making auscultation difficult. The primary advantage of the built-in ANC is that StethAid users can auscultate with a high signal-to-noise ratio, which can be crucial for successful classification. In addition, StethAid features digital filtering to accentuate specific frequency ranges. StethAid also supports a wideband mode in which no filtering is applied. StethAid’s combination of ANC and digital filtering produces a high-quality signal with minimal noise. 

### 3.8. StethAid iOS App

**Figure 5:**
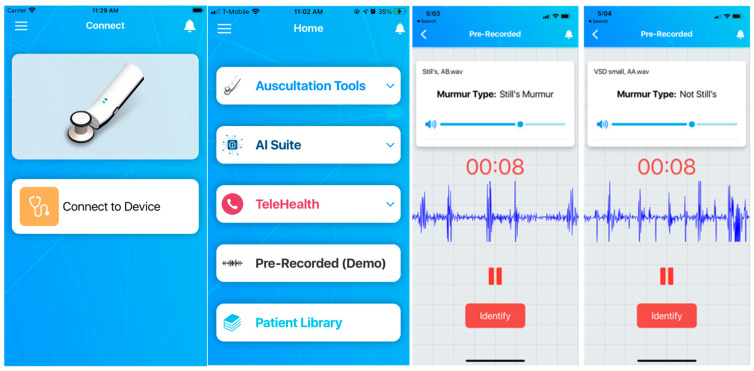
StethAid app features Auscultation Tools, AI Suite, Telehealth, and Patient Library.

The StethAid stethoscope comes with a mobile application which can be used for different purposes such as traditional auscultation using Auscultation Tools, AI suite, Telehealth, and Patient Library (see Figure 5). The app pairs with the device using Bluetooth. The app allows gain setting, enabling/disabling ANC, firmware update, volume control, mic gain setting, and enables auscultation tools such as streaming or recording. StethAid could be used to auscultate lung/heart sounds and voice recordings at the common chest locations. AI Suite runs our deep learning algorithms. The app also allows telehealth. Patient Library stores the recordings locally in the app. The StethAid iOS app is available in the App Store.

### 3.9. StethAid Web Portal

**Figure 6:**
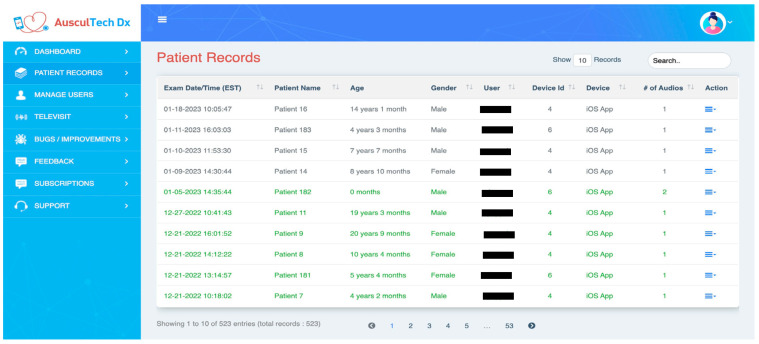
Web portal.

We have developed a web portal connected to the StethAid app that allows clinicians to access and label recorded heart and lung sounds. Each user can securely login to the web portal and access only their associated patient data. When a clinician saves a recording, the heart/lung sound recording and the associated metadata (randomized patient ID, age, gender, physician, and date of visit) are pushed to the remote server. The recordings and the metadata are displayed on the web portal through which the physician can access their patient data (see Figure 6). The physicians can also review, label, and comment on the quality of their associated patient recordings. 

### 3.10. StethAid Telehealth

**Figure 7:**
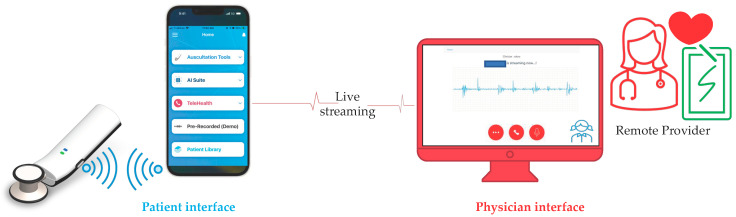
StethAid telehealth patient and physician interfaces.

The StethAid platform features telehealth. With the StethAid digital stethoscope, patients can listen to their own lung and heart sounds and stream them in real time to a secure StethAid portal. The ability to stream cardiopulmonary sounds in real time allows healthcare providers to auscultate and provide a diagnosis even when the patient is not present with them. Figure 7 shows the telehealth workflow in which the StethAid app can be interfaced with the StethAid stethoscope, and the patient can stream auscultated sounds that the provider can visualize and listen to through the StethAid portal.

### 3.11. StethAid Characterization Methodology

**Figure 8:**
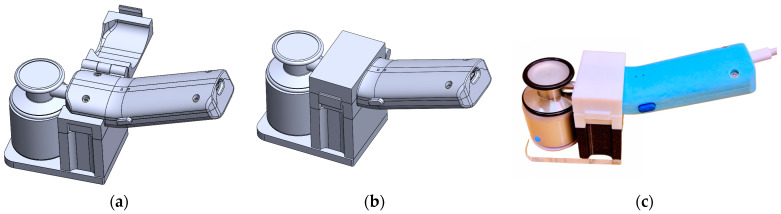
StethAid frequency response measurement rig: (**a**) 3D model showing the rig as open, (**b**) 3D model showing the rig as closed, and (**c**) the StethAid stethoscope mounted on the 3D-printed rig in contact with the speaker.

To determine the frequency response of the StethAid stethoscope, we used a characterization methodology that consists of playing pseudo white noise through a speaker and recording an audio file, from which the frequency response is derived. The complete methodology is described in [24]. For consistent objective frequency response estimation, we designed a measurement rig (see Figure 8). This rig enables the StethAid chestpiece to be placed in contact with the speaker in a consistent reproducible manner. 

## 4. Results

To validate the StethAid platform, we first present a technical characterization of the StethAid stethoscope and the concordance between findings of bedside providers using acoustic stethoscopes and expert physicians reviewing StethAid recordings offline. We then present two example applications of StethAid: Still’s murmur identification and wheeze detection. Both applications include deep learning algorithms trained on datasets collected using StethAid. 

### 4.1. Frequency Response of StethAid Stethoscope

**Figure 9:**
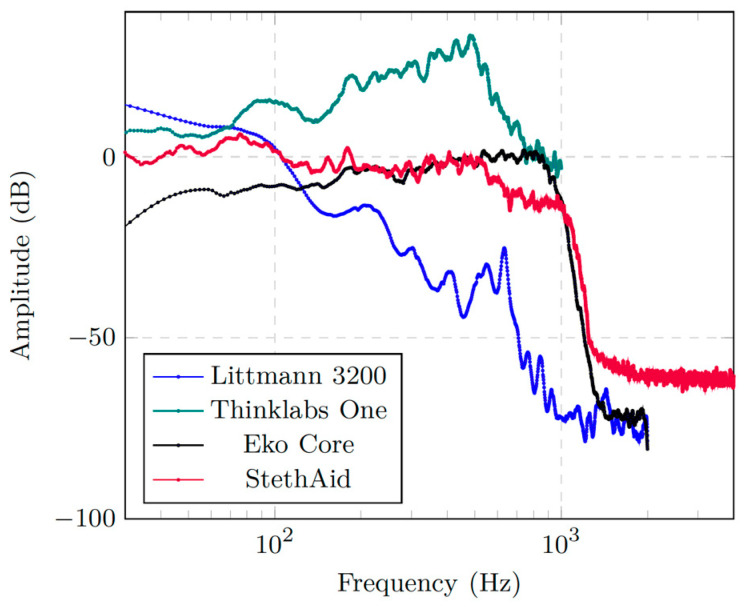
Frequency response of StethAid compared with those of Littmann 3200, Thinklabs One, and Eko Core.

Figure 9 shows the frequency response of the StethAid digital stethoscope versus those of Eko Core, Thinklabs One, and Littmann 3200. The figure shows that the StethAid stethoscope has a response that starts around 0 dB and remains flat for frequencies below 500 Hz. It then decreases slightly for frequencies between 500 and 1000 Hz before it drops to −60 dB for frequencies above 1000 Hz. In general, one can see that the StethAid frequency response is closer to those of Littmann 3200 and Thinklabs One in the low-frequency range (<150 Hz) and tracks that of Eko Core in the 100–1000 Hz range. For frequencies above 1000 Hz, each device sees a sharp drop in gain. Compared with StethAid, Littmann 3200 and Eko Core have slightly lower gains in this range.

### 4.2. Clinical Validation: Concordance between Bedside Providers and Remote Expert Physicians

The goal of this clinical validation was to compare the findings of bedside providers using their acoustic stethoscopes (i.e., bedside labels) with those provided by expert physicians reviewing StethAid recordings offline (i.e., offline labels). In the first study, we determined the concordance between the bedside labels provided by two pediatric cardiologists (RLG and JWM) and the offline labels provided by a third pediatric cardiologist (RWD). We conducted this comparison in 120 participants: 86 with Still’s murmurs and 34 with pathological heart murmurs. The offline labels agreed with the bedside labels in all 86 recordings of Still’s murmurs resulting in a 100% concordance. For pathological heart murmurs, the concordance was 94.1% in that 32 offline labels were the same as the original bedside labels. Two recordings, labeled originally as pathological murmurs at bedside, were labeled as not pathological during offline review. The two pathological heart murmurs were low gradient pulmonary artery stenosis murmurs with trivial severity. The overall concordance was 98.3%.

In the second study, trained research assistants (RAs) recorded lung sounds using StethAid and obtained an ED provider’s exam results on the presence or absence of wheeze. We conducted this study with 29 recordings. Of the 29 recordings, 9 had wheeze and 20 had no wheeze according to the provider using their acoustic stethoscope. Subsequently, three expert physicians (SJP, JCS, and DKP) provided an offline label for these recordings as well as reviewed the sound quality of these recordings. The majority vote between the three expert physicians was considered the final offline label. The quality of the recordings was judged as 28 good recordings and 1 bad/unsure. The expert physicians agreed with the bedside label in 23 cases (79.3%) and disagreed with 6 labels (20.7%). The provider in five of the six disagreement cases heard a wheeze, whereas the expert physicians marked them as clear. The remaining case was marked as unsure by expert physicians and was marked clear by the bedside provider. The 20.3% discordance observed in the recordings could be attributed to several factors, such as temporal disparities between recording acquisition and clinical evaluation, and variations in acquisition protocols. Notably, two recordings were obtained at 16:27, and labels were assigned (before recording) by the provider at 15:35, a considerable time difference which could have resulted in suppressed wheezing sounds if the patient received medication. In contrast, the remaining four recordings were obtained within an average of 5 min from the clinical evaluation in the ED room. However, some of these recordings were flagged as having loud ambient noise, which could account for the discrepancy observed.

### 4.3. StethAid and AI-Based Still’s Murmur Identification

Still’s murmur is the most common innocent murmur in children. It is often difficult to recognize by PCPs [12,14,25]. We are conducting a multicenter data acquisition to create the necessary training and validation sets to develop an automated Still’s murmur identification algorithm. The participating cardiologists perform the murmur recording and provide a diagnosis for it based on the current clinical standard. We have collected 470 recordings. Each recording is 15 s long and is recorded at the lower left sternal border (LLSB) chest location [25]. The dataset consists of 265 Still’s murmur recordings and 205 pathological heart murmur recordings. Each heart sound recording is filtered, denoised, and segmented using our segmentation algorithm [26]. The identified heart cycles are converted to spectrograms, which form the input of a convolutional neural network (CNN). The deep learning architecture is a five-layer CNN that consists of an input layer, three convolution layers, two hidden layers, and an output layer. Each of the three convolution layers consists of a two-dimensional convolution “ReLU” activation function and max pooling. Following the convolution layers, the model has two fully connected layers, each of which has 64 units with “ReLU” activation function. The output layer is a single-neuron layer with a sigmoid activation function. The architecture has been optimized using a Keras Tuner to find the hyperparameters of the CNN. Prior to the segmentation step, we divided the dataset into 70%, 10%, and 20% subsets for training, validating, and testing, respectively. To validate the model, the sensitivity and specificity are reported by recordings, obtained with a majority vote on labels of individual cycles. The developed architecture (CNN-StethAid) achieved 91.9% sensitivity and 92.6% specificity in identifying the Still’s murmur, with an overall accuracy of 92.2% (Table 1). These results are comparable to our previously reported Still’s murmur identification results on a Littmann 4100 dataset (CNN-Littmann 4100) [25]. 

### 4.4. StethAid and AI-Based Wheeze Detection

StethAid has also been deployed in the emergency department (ED) of the Children’s National Hospital to collect lung sound recordings for training and testing a wheeze detection algorithm. We prospectively enrolled children, ages 2 to 18, presenting with asthma exacerbation. RAs utilized the StethAid platform to acquire lung sound recordings together with a provider’s lung exam. Each lung sound recording is 15 s in length and originates from up to 11 different locations on the anterior, posterior, and sides of the chest. Our current wheeze dataset consists of 1095 recordings with provider labels (i.e., bedside labels), of which 644 are clear sounds and 451 are wheeze sounds. These recordings were converted to spectrograms that then formed input to two deep learning models: ResNet-18 and Harmonic Networks. The two models were trained and validated to identify wheeze sounds from clear breath sounds, and the results are presented in Table 2. ResNet18 produced a sensitivity of 77.0%, a specificity of 70.1%, and an accuracy of 73.9%, whereas the Harmonic Networks achieved a sensitivity of 83.7%, a specificity of 84.4%, and an accuracy of 84.0%.

These results demonstrate that accurate identification of wheeze in lung sounds is possible. It should be noted that the performance of our deep learning-based wheeze detection will most likely improve as our dataset grows. 

## 5. Discussions

Table 3 summarizes the features of the available digital stethoscopes against those of StethAid. One can see that Littmann 3200 is an electronic stethoscope that does not have any wireless listening feature, nor does it support any AI-assisted interpretation of the auscultated sounds. Eko Core supports AI for heart murmurs but focuses mostly on adult cardiology. Thinklabs One features heart murmur identification using AI. Feelix is dedicated to asthma and other respiratory diseases and does not presently seem to include any cardiac applications. In comparison, StethAid focuses on pediatrics and features AI algorithms for both cardiac and pulmonary conditions.

Digital auscultation remains an emerging technology, and the list of deficiencies of the currently available digital auscultation platforms can be summarized as follows:

- Variable frequency responses: using multiple stethoscopes to build large-scale, AI-assisted auscultation can be challenging if the frequency characteristics of these stethoscopes are different.
- Limited compatibility/hard to use: Some digital stethoscopes are only compatible with certain software and/or operating systems, which can limit their use in healthcare settings. Some stethoscopes are not intuitive to use, and their sound quality is not superior to that of acoustic stethoscopes, especially in a noisy environment.
- Limited customization: existing digital stethoscopes may not allow for customization of the sound output, which can make it challenging for healthcare professionals with differing hearing abilities or preferences.
- Limited data management: some digital stethoscopes do not have adequate data management systems, making it difficult for healthcare professionals to store and analyze the data collected during auscultation.
- High cost: digital stethoscopes can be expensive, making them inaccessible to some healthcare professionals/patients.
- Limited educational resources: some digital stethoscopes do not come with adequate educational resources to help healthcare professionals learn how to use them effectively.
- Limited telehealth capabilities: some digital stethoscopes do not have adequate telehealth capabilities, making it difficult for healthcare professionals to use them for remote patient care, whether pediatric or adult.

Our digital auscultation platform, StethAid, was designed to aid in the identification of Still’s murmurs using deep learning. While PCPs may be able to detect a murmur when they hear it, their ability to accurately identify the murmur type may be limited. As such, we believe that the development of algorithms to simply detect a murmur does not provide significant clinical value. Additionally, the identification of pathological heart murmurs alone may not be sufficient, as children with such murmurs still require expert consultations with cardiologists to determine the best course of action. We have created a platform that can assist PCPs in distinguishing between an innocent Still’s murmur and potentially pathological non-Still’s murmurs. Such sorting of heart murmurs could reduce the number of unnecessary referrals. There are algorithms, such as eMurmur, that classify pathological murmurs versus innocent murmurs [28]. The eMurmur algorithm’s reported sensitivity and specificity for identifying pathological murmurs are 93% and 81%, respectively, with an overall accuracy of 88%. Our Still’s murmur algorithm achieves 91.9% sensitivity and 92.6% specificity in identifying a Still’s murmur, with an overall accuracy of 92.2%.

Our platform also allows automated wheeze detection in children. Sonavi Labs has created the Feelix digital stethoscope to measure, track, and classify the severity of wheeze. Feelix has also started to focus on other lung conditions [29,30]. A recent paper by Kim et al. [31] reported four algorithms for wheeze detection: 3-layers LSTM, 4-layers CNN, 4-layers CNN+ Tabular data, and ResNet-34, and these models achieved an accuracy of 82.24%, 88.87%, 89.5, and 91.2%, respectively. Our wheeze model’s accuracy is closer to what Kim et al. reported. Bokov et al. [32] reported a support vector machine (SVM)-based wheeze detection that achieved 71.4% sensitivity and 88.9% specificity. The performance of this SVM-based wheeze detection approach was closer to what we reported using our deep learning models. As mentioned, as our dataset grows, we expect the performance of our algorithm to grow as well. 

Finally, our StethAid platform has the capacity to provide telehealth. By enabling telehealth, our platform could have the potential to improve the quality of care for patients with cardiopulmonary conditions, especially in remote or underserved areas where access to healthcare may be limited. Telehealth has provided a convenient alternative for patients who face challenges related to mobility, transportation, and scheduling of in-person visits. Additionally, studies have shown that telehealth provided an effective substitute for in-person visits during the COVID-19 pandemic [33,34], and it is expected to persist in some form beyond the pandemic.

## 6. Conclusions

We developed a digital auscultation platform tailored to pediatrics that consists of a wireless stethoscope, a mobile application, and custom patient portals interacting with cloud storage for centralized data collection. The digital stethoscope has a frequency response comparable to that of three FDA-approved, commercially available stethoscopes. The platform is used for building large pediatric datasets of heart and lung sounds, which, in turn, are being used for developing AI-assisted auscultation tools. We validated our StethAid digital auscultation platform technically and clinically. Our future research directions are the continued research and development of the platform and AI applications. 

## Figures and Tables

**Table 1:** Still’s murmur identification results.

Model	Performance Metrics		
	Sensitivity (%)	Specificity (%)	Accuracy (%)
CNN-Littmann 4100 [25]	90.0	98.3	94.2
CNN-StethAid	91.9	92.6	92.2

**Table 2:** Results of deep-learning wheeze detection.

Model	Performance Metrics		
	Sensitivity (%)	Specificity (%)	Accuracy (%)
ResNet-18 [27]	77.0	70.1	73.9
Harmonic Networks [27]	83.7	84.4	84.0

**Table 3:** Comparison of the features of StethAid and FDA approved digital stethoscopes.

Platform	Digital/Electronic Stethoscope	Wireless Charging	On-Device Recordings	Wireless	AI	Heart Murmurs	Asthma	Pediatrics
Littmann 3200	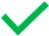	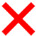	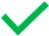	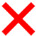	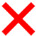	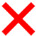	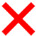	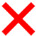
Stethee Pro	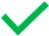	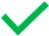	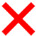	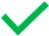	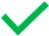	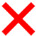	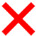	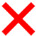
Feelix/Feelix Pro	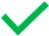	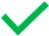	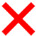	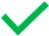	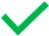	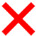	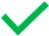	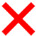
Eko Core	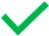	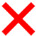	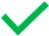	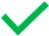	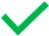	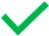	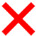	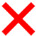
Thinklabs One	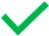	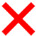	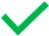	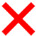	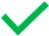	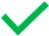	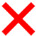	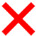
ViScope	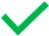	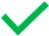	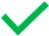	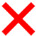	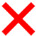	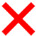	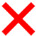	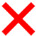
StethAid	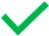	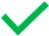	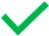	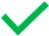	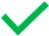	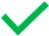	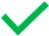	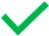

## Data Availability

The data that support the findings of this study are available upon request. All requests should be addressed to the corresponding author.

## References

[1] Roguin A. (2006). Rene Theophile Hyacinthe Laënnec (1781–1826): The man behind the stethoscope. Clin. Med. Res..

[2] Montinari M.R., Minelli S. (2019). The first 200 years of cardiac auscultation and future perspectives. J. Multidiscip. Healthc..

[3] Vasudevan R.S., Horiuchi Y., Torriani F.J., Cotter B., Maisel S.M., Dadwal S.S., Gaynes R., Maisel A.S. (2020). Persistent Value of the Stethoscope in the Age of COVID-19. Am. J. Med..

[4] Weiss D., Erie C., Butera III J., Copt R., Yeaw G., Harpster M., Hughes J., Salem D.N. (2019). An in-vitro acoustic analysis and comparison of popular stethoscopes. Med. Devices.

[5] Ramanathan A., Zhou L., Marzbanrad F., Roseby R., Tan K., Kevat A., Malhotra A. (2019). Digital stethoscopes in paediatric medicine. Acta Paediatr..

[6] Vukanovic-Criley J.M., Criley S., Warde C.M., Boker J.R., Guevara-Matheus L., Churchill W.H., Nelson W.P., Criley J.M. (2006). Competency in cardiac examination skills in medical students, 508 trainees, physicians, and faculty: A multicenter study. Arch. Intern. Med..

[7] Doroshow R.W., Dorner R., Lyons L., Sestokas J. The murmur library: A data bank of recorded heart sounds in children. Proceedings of the Pediatric Educational Excellence across the Continuum.

[8] Herefoss D.A., Andersen S., Halvorsen P.A., Schirmer H., Reierth E., Melbye H. (2023). Diagnostic accuracy of heart auscultation for detecting valve disease: A systematic review. BMJ Open.

[9] Shanthakumari G., Priya E. Spectrogram-based detection of crackles from lung sounds. Proceedings of the 2022 International Conference on Communication, Computing and Internet of Things (IC3IoT).

[10] Kang S., Mcconnaughey J., Doroshow R., Shekhar R. (2015). Automated Recognition of Still’s Murmur in Children. IEEE Trans. Biomed. Eng..

[11] Oort A.V., Blanc-Botden M.L., Boo T.D., Werf T.V.D., Rohmer J., Daniëls O., Van Oort A., Blanc-botden M.L., Boo T.D., Werf T.V.D. (1994). The Vibratory Innocent Heart Murmur in Schoolchildren: Difference in Auscultatory Findings between School Medical Officers and a Pediatric Cardiologist. Pediatr. Cardiol..

[12] Kang S., Doroshow R., McConnaughey J., Shekhar R. (2017). Automated Identification of Innocent Still’s Murmur in Children. IEEE Trans. Biomed. Eng..

[13] Zahran H.S., Bailey C.M., Damon S.A., Garbe P.L., Breysse P.N. (2018). Vital Signs: Asthma in Children—United States, 2001–2016. MMWR Morb. Mortal. Wkly. Rep..

[14] Koonin L.M., Brooke H., Clarisse A., Tsang Z.L., Kevin F., Brandon J., Peter A., McCabe B., Zelis C.B.R., Tong I. (2020). Trends in the use of telehealth during the emergence of the COVID-19 pandemic—United States, January–March 2020. Morb. Mortal. Wkly. Rep..

[15] Leng S., Tan R.S., Chai K.T., Wang C., Ghista D., Zhong L. (2015). The electronic stethoscope. Biomed. Eng. Online.

[16] 3M Littmann Anatomy of a Stethoscope. www.littmann.com.

[17] Oliynyk V. (2013). Determination of the amplitude-frequency characteristic of the 3M Littmann 3200 Digital stethoscope. Acoust. Bull..

[18] StetheePro Tech Specs (M3dicine, 2023). https://m3dicine.com/products/stethee-pro/stethee-pro-tech-specs/.

[19] Feelix. https://sonavilabs.com/feelix-pro/.

[20] 3M™ Littmann® CORE Digital Stethoscope. https://shop.ekohealth.com/products/3m-littmann-core-digital-stethoscope?variant=39307014209632.

[21] ThinkLabs One Stethoscope (2023). https://www.thinklabs.com.

[22] ViScope. https://www.medicalexpo.com/prod/hd-medical-group/product-95579-592845.html.

[23] Arjoune Y., Salvador T., Nguyen T.N., Telluri A., John T., Schroder J., Pillai D., Teach S., Patel S., Doroshow R.W. (2023). Stethaid: An Electronic Stethoscope Connected to iOS Mobile Apps for AI-Assisted Auscultation. Proceedings of the 2023 Design of Medical Devices Conference.

[24] Arjoune Y., Nguyen T.N., Doroshow R.W., Shekhar R. (2023). Technical Characterisation of Digital Stethoscopes: Toward Scalable Artificial Intelligence-Based Auscultation. J. Med. Eng. Technol..

[25] Shekhar R., Vanama G., John T., Issac J., Arjoune Y., Doroshow R.W. (2022). Automated identification of innocent Still’s murmur using a convolutional neural network. Front. Pediatr..

[26] Arjoune Y., Nguyen T.N., Doroshow R.W., Shekhar R. (2022). A Noise-Robust Heart Sound Segmentation Algorithm Based on Shannon Energy and Smart Cropping. Submitted to IEEE J. Biomed. Health Inform..

[27] Nguyen T.N., Arjoune Y., Schroder J.C., Pillai D.K., Patel S.J., Shekhar R. Machine Learning for Automated Wheeze Detection in Children. Proceedings of the 2022 IEEE International Conference on Big Data (Big Data).

[28] Thompson W.R., Reinisch A.J., Unterberger M.J., Schriefl A.J. (2019). Artificial intelligence-assisted auscultation of heart murmurs: Validation by virtual clinical trial. Pediatr. Cardiol..

[29] Patel S.J., Arnold D.H., Topoz I., Sills M.R. (2018). Literature review: Prediction modeling of emergency department disposition decisions for children with acute asthma exacerbations. Clin. Pediatr. Emerg. Med..

[30] Patel S.J., Chamberlain D.B., Chamberlain J.M. (2018). A Machine Learning Approach to Predicting Need for Hospitalization for Pediatric Asthma Exacerbation at the Time of Emergency Department Triage. Acad. Emerg. Med..

[31] Kim B.J., Kim B.S., Mun J.H., Lim C., Kim K.H. (2022). An accurate deep learning model for wheezing in children using real world data. Sci. Rep..

[32] Bokov P., Mahut B., Flaud P., Delclaux C. (2016). Wheezing recognition algorithm using recordings of respiratory sounds at the mouth in a pediatric population. Comput. Biol. Med..

[33] Kuhn L., Reeves K., Taylor Y. (2015). Planning for Action: The Impact of an Asthma Action Plan Decision Support Tool Integrated into an Electronic Health Record (EHR) at a Large Health Care System. J. Am. Board Fam. Med..

[34] Shaver J. (2022). The State of Telehealth Before and After the COVID-19 Pandemic. Prim. Care.

